# Considerations for Oral Cholera Vaccine Use during Outbreak after Earthquake in Haiti, 2010–2011

**DOI:** 10.3201/eid1807.120071

**Published:** 2012-07

**Authors:** Lorenz von Seidlein, Jacqueline L. Deen

**Affiliations:** Menzies School of Health Research, Casuarina, Northern Territory, Australia

**Keywords:** cholera outbreak, cholera vaccination, oral cholera vaccine (OCV), bacteria, disaster, post-earthquake Haiti, waterborne infections, Haiti

**To the Editor:** We wish to thank Date et al. for their clear discussion of the arguments against the use of oral cholera vaccines (OCVs) in Haiti in 2010–11 (*1*). The epidemic curve in their article suggests that the control activities had an effect on mortality rates, resulting in a decrease in case-fatality rates to <1%. This finding is a remarkable success not achieved during the recent cholera outbreak in Zimbabwe that affected 98,531 persons, of whom 4,282 (4.3%) died (*2*). However, the article does not discuss the lack of effect of the control measures in Haiti on the spread of the epidemic. Considering the failure of containment, it would have been interesting to read how the authors judge the recommendation not to vaccinate, with the benefit of hindsight.

The authors list a catalog of arguments against the use of OCVs in outbreaks. These included the priority of water provision and cholera treatment measures, how modeling data provided no convincing justification for vaccination campaigns, how mobile populations cannot be trusted to take 2 doses, the time for a 2-dose vaccine to generate immunity, the logistic challenges in a setting of inadequate infrastructure and human resources, the cold chain requirements, the difficulty in transport of bulky vaccine, clean water requirements for the buffer, civil unrest, and an unpredictable response from the public.

Overall, we agree entirely that a mass cholera vaccination campaign is a massive logistic challenge. We do, however, question whether logistic challenges of similar size would stop vaccination campaigns against, e.g., influenza in Hong Kong, People’s Republic of China. We are convinced that citizens of Hong Kong and their advocates would not tolerate such arguments regarding challenges. Is it because the at-risk population in Haiti is perceived to have few, if any, powerful advocates that such arguments listed by the Centers for Disease Control and Prevention and the Pan American Health Organization could be applied unchallenged?

A much stronger argument against vaccinations is the limited availability of an appropriate licensed vaccine prequalified for purchase by United Nations agencies. At the start of the outbreak October 2010, only 1 OCV, Dukoral (Crucell, Leiden, the Netherlands), was licensed and prequalified. However, not even 300,000 doses of Dukoral were available at the start of the outbreak. A second OCV, Shanchol (Shantha Biotechnics Ltd., Basheerbagh,

Hyderabad, India), was licensed but was prequalified only in September 2011. The bigger question is why the international agencies failed to ensure an appropriate vaccine supply following the catastrophic cholera outbreak in Zimbabwe in 2008–2009. Highly effective OCVs have been licensed since 1991 and are marketed to affluent tourists who are at little, if any, risk of being exposed to cholera. The neglect of OCVs as a public health tool during the past 20 years represents a failure of the cholera experts and policymakers alike. Again, such a failure would be unthinkable for a disease affecting more privileged population groups.

The authors write that the lack of data proving that reactive vaccination campaigns are effective was an argument against the use of OCVs in Haiti. We are in agreement that it is unknown whether a reactive mass cholera vaccination campaign would result in adequate vaccine coverage to provide protection and contain further spread. There are simply no data. It is surprising that the Centers for Disease Control and Prevention and Pan American Health Organization experts did not recognize and use the unique opportunity in Haiti to conduct mass vaccination campaigns for the purpose of collecting such vital data.

Finally, the argument of questionable cost-effectiveness is mentioned by the authors. Indeed, data are lacking on the economic benefits of using OCVs in severe outbreaks, although their cost-effectiveness in cholera-endemic situations has been demonstrated (*3*). We believe that anyone who has lived through the agonizing indignities of a cholera attack, especially during a cholera outbreak, would dismiss the economic argument out of hand. No one should have to suffer, much less to die from a vaccine-preventable and quickly curable disease. Using the argument that vaccinations could be too expensive is morally questionable, if not to say revolting.

We have arrived at the conclusion that the withholding of cholera vaccines during the outbreak in Haiti in 2010–2011 was a judgment error and missed opportunity to collect useful data. We wonder whether this article was written to justify what turns out to be an unsound decision, considering the move by other agencies to proceed with a pilot cholera vaccination campaign (*4*). We believe that persistent neglect of OCV as a public health tool is based on the shortcomings of the current generation of cholera experts and policy makers. The long list of technical reasons provided by the authors regarding why the implementation of mass vaccinations was impossible in Haiti are plausible excuses. However, the true reason that cholera vaccines have not been used in Haiti 20 years after they have been licensed and shown to be effective is the fact that populations affected by cholera outbreaks are underprivileged, even by the standards of impoverished populations. It will take decision makers who are less risk-averse and more compassionate to contain the next cholera outbreak. We hope that future decisions will not be biased by previous untrue dogma that vaccination and other measures such as sanitation and effective treatment would oppose each other when the opposite is true. A more enlightened environment would enable more widespread use of OCVs.
