# Supplementary material for: Considerations for Oral Cholera Vaccine Use during Outbreak after Earthquake in Haiti, 2010–2011
Source: Emerg Infect Dis. 2012 Jul;18(7):1211–4. doi: 10.3201/eid1807.120071 (PMC3376809; doi:10.3201/eid1807.120071)
Supplement: Related texts

# Reply

**DOI:** 10.3201/eid1807.120408

**Authors:** Kashmira Date, Terri Hyde, Eric Mintz, Andrea Vicari, M. Carolina Danovaro-Holliday, Cuauhtemoc Ruiz-Matus, Ariel Henry, Jon Andrus, Vance Dietz

**Affiliations:** Centers for Disease Control and Prevention, Atlanta, Georgia, USA (K. Date, T. Hyde, E. Mintz., V. Dietz);; Pan American Health Organization, Washington DC, USA (A. Vicari, M.C. Danovaro-Holliday, C. Ruiz-Matus, J. Andrus);; Formerly, Haitian Ministry of Population and Public Health, Port-au-Prince, Haiti (A. Henry)

**In Response:** Drs. von Seidlein and Deen criticized decisions regarding oral cholera vaccine (OCV) use in Haiti, but acknowledged that there are no data showing that a reactive mass OCV campaign would contain further disease spread (*1*). They agreed that such a campaign is a massive logistic challenge and asserted that the limited supply of World Health Organization (WHO)–prequalified OCV available during the first 11 months of the epidemic in Haiti was an even stronger argument against vaccination. They then asserted that: 1) by “withholding” “highly effective” OCVs during the outbreak in Haiti, an opportunity “to collect data” was missed; 2) the decision not to vaccinate against cholera “was tolerated” because, like other economically disadvantaged populations, “Haitians have few powerful advocates;” and, 3) the limited OCV supply represents a “failure of the current generation of cholera experts and policymakers.”

In our institutions’ efforts to support national authorities, the welfare of the Haitian people was and remains our primary concern. Our publication describes considerations during the initial response to an expanding epidemic, when the focus was on saving lives; recommendations were revisited after immediate cholera treatment and prevention efforts were successfully established.

As documented in our report (*2*) and in the media (*3**,**4*), the decision at the peak of the epidemic to not use the available doses of WHO-prequalified OCV to vaccinate 150,000 persons (1.5% of the Haitian population) was made by the Haitian government, in the setting of well-publicized differences of opinion among experts. Although this decision may have resulted in “data not being collected,” decisions by sovereign governments are rarely overruled by international organizations, scientists or policymakers. Vaccine use without government approval would have raised questions about the appropriateness of using the outbreak to pilot a large-scale reactive cholera vaccination campaign without documented effectiveness.

OCV effectiveness is moderate when compared with measles and rubella vaccines. Although vaccine-preventable diseases, e.g., measles and influenza, are primarily prevented through vaccination, cholera can be prevented and controlled through other means.

It is true that underprivileged, impoverished populations are disproportionately affected by epidemic cholera, as they have been for centuries. This is not for lack of access to OCVs (which are also unavailable in the United States), but because of lack of access to potable water and adequate sanitation. OCVs do have a place in cholera prevention and response, but a greater public health deficit underlies the spread of cholera in Haiti and other countries where it remains endemic or epidemic. Ensuring universal access to safe water and sanitation, beyond recent progress toward meeting the Millennium Development Goals (*5*), is vital for global cholera control. The Pan American Health Organization (PAHO), the United Nations Children’s Fund, and the Centers for Disease Control and Prevention (CDC) have called upon the international community to assist Haiti in this effort (*6*).

The issue of OCV availability is being addressed by various public health organizations. In September 2011, WHO convened an expert consultation to discuss the strategic framework for an OCV stockpile (*7*); the second follow-up meeting was planned for April 26–27, 2012, for further action. Recently, the Coalition for Cholera Prevention and Control, funded by the Bill & Melinda Gates Foundation, held an inaugural meeting of cholera and immunization experts and policymakers to develop comprehensive cholera prevention and control strategies that include appropriate use of OCVs in endemic and epidemic settings (*8*). Cholera outbreaks are unpredictable; increased demand from endemic countries can ultimately drive vaccine production, and help maintain a stockpile for outbreak use.

During the past 20 years, a substantial effort has been made by CDC, WHO, PAHO, and private and public partners working with governments to provide existing vaccines in an equitable manner to some of the world’s most disadvantaged populations, and to ensure that these populations have equal opportunities to receive new vaccines, such as rotavirus and pneumococcal vaccines (*9**,**10*). New vaccines require greater investments than in the past; criteria such as preventable burden, cost-effectiveness, and sustainability are key to systematic, evidence-based vaccine introductions (*11**,**12*). CDC and PAHO are providing technical and financial assistance to the Haitian government for improving the national vaccine cold chain capacity; launching a measles, rubella and polio catch-up campaign; and introducing pentavalent (diphtheria + tetanus + pertussis + *Haemophilus influenzae* type b + hepatitis B), rotavirus, and pneumococcal vaccines. With approval of the Haitian government, CDC and PAHO have provided technical assistance to 2 organizations implementing small-scale OCV campaigns in Haiti.

Contrary to the authors’ suggestion of “unquestioned dogma,” the current generation of cholera and immunization experts and policymakers are engaged in developing an evidence-based, integrated approach to cholera prevention and control that will optimize OCV use without neglecting either primary prevention through improvements in water, sanitation, and hygiene, or prevention of cholera-related deaths through improved access to life-saving treatment. All cholera prevention and control measures for populations at highest risk need the continued support of powerful advocates in the scientific, political, and policy-making spheres.

## References

[1] von Seidlein L, Deen JL. Considerations for oral cholera vaccine use during outbreak after earthquake in Haiti, 2010–2011 [letter]. Emerg Infect Dis. 2012;18:1211–12.2270942510.3201/eid1807.120071PMC3376809

[2] Date KA, Vicari A, Hyde TB, Mintz E, Danovaro-Holliday MC, Henry A, Considerations for oral cholera vaccine use during outbreak after earthquake in Haiti, 2010–2011. Emerg Infect Dis. 2011;17:2105–12. 10.3201/eid1711.11082222099114PMC3310586

[3] Cyranoski D. Cholera vaccine plan splits experts. Nature. 2011;469:273–4. 10.1038/469273a21248807

[4] Jack A. Haiti refused cholera vaccine, chief says [cited 2012 Mar 6]. Financial Times. July 20, 2011 [cited 2012 Mar 6]. http://www.ft.com/intl/cms/s/0/323038a4-ab24-11e0-b4d8-00144feabdc0.html

[5] United Nations Children’s Fund and World Health Organization Joint Monitoring Programme for Water Supply and Sanitation. Progress on drinking water and sanitation, March 6 2012 [cited 2012 Mar 6]. http://www.who.int/water_sanitation_health/monitoring/jmp2012/en/index.html

[6] Periago MR, Frieden TR, Tappero JW, De Cock KM, Assen B, Andrus JK. Elimination of cholera transmission in Haiti and the Dominican Republic. Lancet. 2012;379:e12–3. 10.1016/S0140-6736(12)60031-222240408

[7] World Health Organization. WHO consultation on oral cholera vaccine (OCV) stockpile strategic framework: potential objectives and possible policy options (draft report). Geneva: The Organization; 2011 [cited 2012 Mar 6]. http://www.who.int/water_sanitation_health/monitoring/jmp2012/en/index.html

[8] The Taskforce for Global Health. The Task Force for Global Health and Partners in Health to convene coalition for cholera prevention and control, December 7, 2011 [cited 2012 Mar 6]. http://www.taskforce.org/press-room/press-releases/task-force-global-health-and-partners-health-convene-coalition-cholera-pre

[9] Global Alliance for Vaccines Initiative. Vaccines against major childhood diseases to reach 37 more countries, September 27, 2011 [cited 2012 Mar 9]. http://www.gavialliance.org/library/news/press-releases/2011/vaccines-against-major-childhood-diseases-to-reach-37-more-countries

[10] Andrus JK, Crouch AA, Fitzsimmons J, Vicari A, Tambini G. Immunization and the Millennium Development Goals: progress and challenges in Latin America and the Caribbean. Health Aff (Millwood). 2008;27:487–93. 10.1377/hlthaff.27.2.48718332506

[11] World Health Organization. Vaccine introduction guidelines. Adding a vaccine to a national immunization programme: decision and implementation, November 2005. Geneva: The Organization; 2005 [cited 2012 Mar 6]. http://www.who.int/vaccines-documents/DocsPDF05/777_screen.pdf

[12] World Health Organization. Global plan of action for new and under-utilized vaccines implementation: 2010–2011, July 28, 2010. Geneva: The Organization; 2010 [cited 2012 Mar 6]. http://www.who.int/nuvi/2010_07_28_NUVI_PoA_2010-2011.pdf
